# Site I_Q_ in mitochondrial complex I generates S1QEL-sensitive superoxide/hydrogen peroxide in both the reverse and forward reactions

**DOI:** 10.1042/BCJ20220611

**Published:** 2023-03-15

**Authors:** Edwin T. Gibbs, Chad A. Lerner, Mark A. Watson, Hoi-Shan Wong, Akos A. Gerencser, Martin D. Brand

**Affiliations:** Buck Institute for Research on Aging, 8001 Redwood Blvd., Novato, CA 94945, U.S.A.

**Keywords:** complex I, electron transport chain, mitochondria, reactive oxygen species, reverse electron transport, S1QEL

## Abstract

Superoxide/hydrogen peroxide production by site I_Q_ in complex I of the electron transport chain is conventionally assayed during reverse electron transport (RET) from ubiquinol to NAD. However, S1QELs (specific suppressors of superoxide/hydrogen peroxide production by site I_Q_) have potent effects in cells and *in vivo* during presumed forward electron transport (FET). Therefore, we tested whether site I_Q_ generates S1QEL-sensitive superoxide/hydrogen peroxide during FET (site I_Q_f), or alternatively, whether RET and associated S1QEL-sensitive superoxide/hydrogen peroxide production (site I_Q_r) occurs in cells under normal conditions. We introduce an assay to determine if electron flow through complex I is thermodynamically forward or reverse: on blocking electron flow through complex I, the endogenous matrix NAD pool will become more reduced if flow before the challenge was forward, but more oxidised if flow was reverse. Using this assay we show in the model system of isolated rat skeletal muscle mitochondria that superoxide/hydrogen peroxide production by site I_Q_ can be equally great whether RET or FET is running. We show that sites I_Q_r and I_Q_f are equally sensitive to S1QELs, and to rotenone and piericidin A, inhibitors that block the Q-site of complex I. We exclude the possibility that some sub-fraction of the mitochondrial population running site I_Q_r during FET is responsible for S1QEL-sensitive superoxide/hydrogen peroxide production by site I_Q_. Finally, we show that superoxide/hydrogen peroxide production by site I_Q_ in cells occurs during FET, and is S1QEL-sensitive.

## Introduction

There is extensive evidence that mitochondria generate superoxide and/or hydrogen peroxide at substantial rates in intact cells and *in vivo*, and that this superoxide/hydrogen peroxide is important in both physiological redox signalling and pathological signalling, damage and disease [1]. Studies using isolated mitochondria have identified at least 11 different sites of superoxide/hydrogen peroxide production within the mitochondrial electron transport chain and associated dehydrogenases, and have characterised their maximum rates and the conditions needed to achieve them [2,3]. Three sites are of most relevance here. Site I_F_ is the site of superoxide/hydrogen peroxide production in respiratory complex I that (in intact mitochondria) is dependent only on the redox state of the flavin. It is conventionally measured during forward electron transport (FET) in the thermodynamically-favoured direction from NAD-linked substrates such as glutamate plus malate (G + M) to ubiquinone (Q) in the presence of Q-site inhibitors of complex I such as rotenone or piericidin A to keep the flavin very reduced. Site I_Q_ is the site of superoxide/hydrogen peroxide production in complex I that is dependent on the redox states of the flavin and particularly of the quinone and on the components of the protonmotive force. It is conventionally measured (as site I_Q_r) during reverse electron transport (RET) in the thermodynamically unfavoured direction (i.e. requiring energy input from the protonmotive force) from the Q pool into complex I driven by succinate oxidation, which generates the required conditions of a strongly reduced QH_2_/Q ratio together with a high protonmotive force and a high transmembrane pH gradient. Site III_Qo_ is the site of superoxide production at the outer quinone-binding site of respiratory complex III. It is conventionally measured in the presence of sufficient succinate to reduce the Q pool only partially, and of the Q_i_-site inhibitor antimycin A to inhibit onward electron flow. The characterisation of these sites enabled the discovery of site-specific small molecules that suppress superoxide/hydrogen peroxide production without affecting normal electron transport: suppressors of site I_Q_ electron leak (S1QELs) [4,5], and suppressors of site III_Qo_ electron leak (S3QELs) [6].

The molecular mechanism by which S1QELs suppress superoxide/hydrogen peroxide generation at site I_Q_ remains unproven; our current model [3] has S1QELs binding to complex I to alter rate constants at the Q-binding site to decrease electron flow from a semiquinone directly to oxygen without affecting electron flow to and from the QH_2_/Q pool. Others [7–9] have proposed that S1QELs work solely by inhibiting RET from QH_2_ into complex I. However, we have shown that S1QELs suppress superoxide/hydrogen peroxide production at much lower concentrations than they inhibit RET, and therefore we reject that hypothesis [10].

Superoxide/hydrogen peroxide production by site I_Q_ is conventionally assayed during RET, i.e. as site I_Q_r [2,3,11]. To generate superoxide/hydrogen peroxide at high rates site I_Q_ needs a strongly reduced QH_2_/Q ratio [8,12,13], high protonmotive force and high ΔpH [12,14]. Despite contrary claims [8,15] there is good evidence that site I_Q_ is functionally different from site I_F_ [3,10,13,14,16]. Under conventional conditions of saturating substrate concentrations with no added ADP and no added inhibitors, site I_Q_ runs in isolated rat muscle mitochondria only during RET from QH_2_ to NAD^+^ with succinate or glycerol 3-phosphate (G3P) as respiratory substrate (i.e. as I_Q_r). It does not run during FET from NADH to Q with excess G + M as substrate (i.e. as I_Q_f) [11,17]. However, under contrived conditions of strongly reduced NADH/NAD^+^ and QH_2_/Q pools, high protonmotive force and high ΔpH in the presence of Q-site inhibitors such as rotenone or piericidin A, it can be shown to run in a stalled and presumably unphysiological I_Q_f mode [14,18].

As determined by the extent of sensitivity of extracellular hydrogen peroxide release to added S1QELs, site I_Q_ runs in cells [19–23], where it contributes ∼15% of total cellular hydrogen peroxide production. Within cells, it is responsible for the majority of superoxide/hydrogen peroxide production in the mitochondrial matrix [21]. These results hold in a variety of cell types from different organs and species [22]. As determined by the sensitivity of pathological phenotypes to the addition of S1QELs, site I_Q_ can also generate pathological amounts of superoxide/hydrogen peroxide *in vivo* [24–26].

These considerations raise an intriguing and important question. S1QELs have potent effects in cells and *in vivo*, yet under physiologically plausible conditions *in vitro* they have only been shown to suppress superoxide/hydrogen peroxide production during RET. Does this mean that RET (and superoxide/hydrogen peroxide production by site I_Q_r) is common in cells and *in vivo* under normal and pathological conditions, or does it mean that site I_Q_ normally runs during forward electron flow as site I_Q_f, yet is still S1QEL-sensitive? Consistent with the first possibility, S1QELs have been shown to protect strongly against ischaemia-reperfusion injury in the Langendorff heart model [5], in which the injury is generally thought to occur through the operation of site I_Q_r during RET upon reoxygenation in the presence of excess accumulated succinate [27,28]. Others conclude that RET occurs in fruit flies and extends lifespan, based on the effects of rotenone inhibition [29–31]. However, the first possibility implies that the conventional description of oxidative phosphorylation under normal conditions from NAD-linked substrates in cells and *in vivo*, involving proton pumping and production of ATP from each of complex I, complex III and complex IV, is often incorrect, with complex I operating in reverse and only complexes III and IV generating ATP, which is not an attractive hypothesis.

To address this question, we introduce here a simple assay of the direction of electron flow through complex I in isolated mitochondria and in cells. We use it to determine whether site I_Q_ can generate superoxide/hydrogen peroxide in both the reverse and forward reactions in the absence of complex I inhibitors, and whether both modes of I_Q_ activity, I_Q_r and I_Q_f, are sensitive to S1QELs.

## Results

### Assay for forward electron transport in isolated mitochondria

The initial aim of this study was to determine if S1QELs suppress superoxide/hydrogen peroxide production from site I_Q_ of the electron transport chain during forward electron flow when electrons are supplied from NADH (i.e. site I_Q_f). The only existing assay of superoxide/hydrogen peroxide production by site I_Q_f [18] employs ATP hydrolysis to maintain protonmotive force, succinate to maintain a high QH_2_/Q ratio, and complex I Q-site inhibitors, such as rotenone, piericidin A, or a high concentration of myxothiazol, to prevent electron escape from complex I during the assay. However, these conditions are unsuitable for the assay of site I_Q_f in intact cells and *in vivo*, where we want to suppress site I_Q_f without preventing normal electron transport from NADH to Q. The Q-site inhibitors may also interact with the binding of S1QELs to complex I [10], making this assay [18] unsuitable for determining the S1QEL-sensitivity of site I_Q_f. Indeed, preliminary experiments (not shown) revealed that S1QELs suppress superoxide/hydrogen peroxide production only weakly or not at all in the Lambert and Brand [18] I_Q_f assay (H.-S. Wong, E.T. Gibbs II and M.D. Brand, unpublished observations).

Therefore, we devised a novel assay of FET and site I_Q_f that does not require the addition of rotenone, piericidin A, or myxothiazol except to establish that FET is running under the defined condition. It is straightforward to run complex I under conditions of FET (by adding excess of a substrate such as G + M, or pyruvate plus malate, to fully reduce the matrix NAD pool) or conditions of RET (by adding a substrate that feeds electrons directly to the Q pool, such as succinate or G3P, to provide both a high QH_2_/Q ratio and a high protonmotive force to drive electron flow in the thermodynamically unfavourable reverse direction through complex I from QH_2_ to NAD). The requirement here is to set up conditions with FET or RET but favouring superoxide/hydrogen peroxide production at site I_Q_, i.e. with high QH_2_/Q ratio, high protonmotive force and high ΔpH (which are usually absent from FET assays with G + M alone [18,32]), and then to show under the specific conditions of the assay whether electron flow is from added G + M to NADH to Q (forward electron transport) or from added succinate or G3P to QH_2_ to NAD^+^ (reverse electron transport). To determine the direction of electron flow we employ a rotenone challenge. If complex I is running FET then the NAD pool will become more reduced when rotenone addition blocks NADH reoxidation through complex I. Conversely, if complex I is running RET then the NAD pool will become more oxidised when rotenone addition blocks the reduction in NAD by QH_2_ through complex I. Once we have established that FET (or RET) is occurring under a specific assay set-up, we can omit the rotenone challenge and determine the rate of superoxide/hydrogen peroxide production from site I_Q_f (or I_Q_r) in that assay condition, and its sensitivity to the addition of S1QELs.

The conventional substrate to drive RET and site I_Q_r is succinate, to maintain a high QH_2_/Q ratio and a high protonmotive force and ΔpH. Under these conditions, even though the NAD pool becomes 90–95% reduced by RET [13,17,18], we would readily see its reoxidation on rotenone challenge, diagnostic of RET. However, to diagnose FET and putative activity of site I_Q_f we would need to poise the reduction state of the NAD pool with succinate (to maintain a high QH_2_/Q ratio and a high protonmotive force and ΔpH) plus a small concentration of G + M (to provide electrons to the NAD pool to drive FET) then observe the NAD pool become even more reduced on rotenone challenge. Preliminary experiments showed that the dynamic range to observe unambiguous further reduction in the NAD pool on rotenone challenge when using succinate as substrate was too limited (i.e. the NAD pool was too strongly reduced by this substrate). Unlike some other mitochondria, such as those from liver, skeletal muscle mitochondria have an adequate activity of the mitochondrial isoform of G3P dehydrogenase (mGPDH) [17,33,34], so we used G3P as substrate to give submaximal NAD reduction but sufficient QH_2_/Q ratio and protonmotive force to support site I_Q_f.

**Figure 1.**
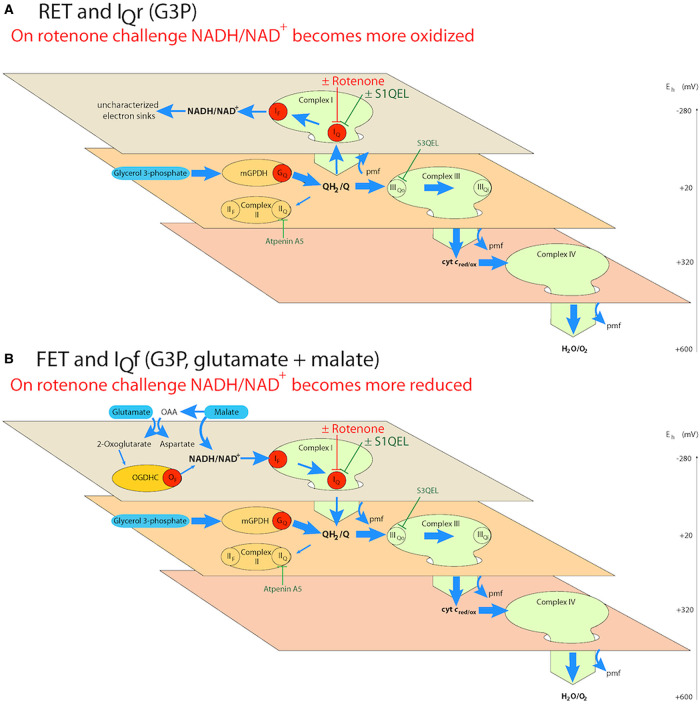
Principle of distinguishing reverse electron transport (RET) from forward electron transport (FET) and assaying superoxide/hydrogen peroxide production from sites I_Q_r and I_Q_f in isolated skeletal muscle mitochondria. Schemes depict the electrochemical topology of the electron transport chain. The three planes represent different isopotential groups within the electron transport chain, with the redox potential, *E*_h_, indicated by the scale. Green ovals represent complexes I, III and IV, red discs represent sites of superoxide/hydrogen peroxide production: sites I_F_ and I_Q_ in complex I, G_Q_ in mitochondrial glycerol 3-phosphate dehydrogenase (mGPDH), and O_F_ in the 2-oxoglutarate dehydrogenase complex (OGDHC) [3]. S1QELs [4,5] can be used to suppress superoxide/hydrogen peroxide production by site I_Q_ in either the reverse or forward reaction (see the text). If required to decrease the background activity of other sites, superoxide production by site III_Qo_ in complex III can be suppressed using S3QELs [6] and electron flow through complex II and into site II_F_ can be blocked using atpenin A5 [36–39]. (**A**) Reverse electron transport and I_Q_r. Oxidation of glycerol 3-phosphate by mGPDH reduces the Q pool, and further oxidation through complexes III and IV (thick blue arrows) generates a high protonmotive force and ΔpH; these conditions drive electrons from QH_2_ upstream into complex I and the NAD pool to set up reverse electron transport (thin blue arrows) and superoxide/hydrogen peroxide production by site I_Q_r. (**B**) Forward electron transport and I_Q_f. The addition of a low but sufficient (titrated) concentration of glutamate plus malate to the conditions of (**A**) trickles electrons into NAD from upstream malate and 2-oxoglutarate dehydrogenases to increase the reduction in the NAD pool, which switches electron flow through complex I from reverse to forward electron transport from NADH to Q. In either (**A**) or (**B**), the subsequent addition of rotenone to block electron flow at the Q-site of complex I (rotenone challenge) will cause the NAD pool to oxidise if reverse electron transport was operative and electron supply was predominantly from QH_2_, but to reduce if forward electron transport was operative and electron supply was predominantly from glutamate plus malate before the challenge, to demonstrate reverse and forward electron transport, respectively. OAA, oxaloacetate.

Figure 1 shows the principle of the novel FET and I_Q_f assay and its use to also demonstrate conventional RET and I_Q_r. If we use G3P alone as the reductant to reduce Q strongly and set up a large protonmotive force and ΔpH by electron flow through complex III and complex IV (Figure 1A), electrons are driven by RET into complex I to reduce the NAD pool. On rotenone challenge, the electron flow from QH_2_ to the NAD pool is abruptly prevented, and electron flow to uncharacterised sinks (such as mitochondrial diaphorases or reversal of other dehydrogenase reactions [35]) allows the NADH to reoxidise slowly, demonstrating RET and the possibility of superoxide/hydrogen peroxide by site I_Q_r. If we use G3P as the reductant to reduce Q strongly and set up large protonmotive force and ΔpH by electron flow through complex III and complex IV, but concurrently add a small concentration of G + M that is just sufficient to drive forward flow from upstream dehydrogenases yet not make the NAD pool too reduced to see further reduction on rotenone challenge (Figure 1B), then electrons are driven by FET through complex I. On rotenone challenge the electron flow from NADH to QH_2_ is abruptly prevented, in this case allowing the NADH to become further reduced, demonstrating FET and the possibility of superoxide/hydrogen peroxide by site I_Q_f.

### Demonstration of reverse and forward electron transport through complex I in isolated rat skeletal muscle mitochondria and superoxide/hydrogen peroxide production from sites I_Q_r and I_Q_f using cuvette-based assays

**Figure 2.**
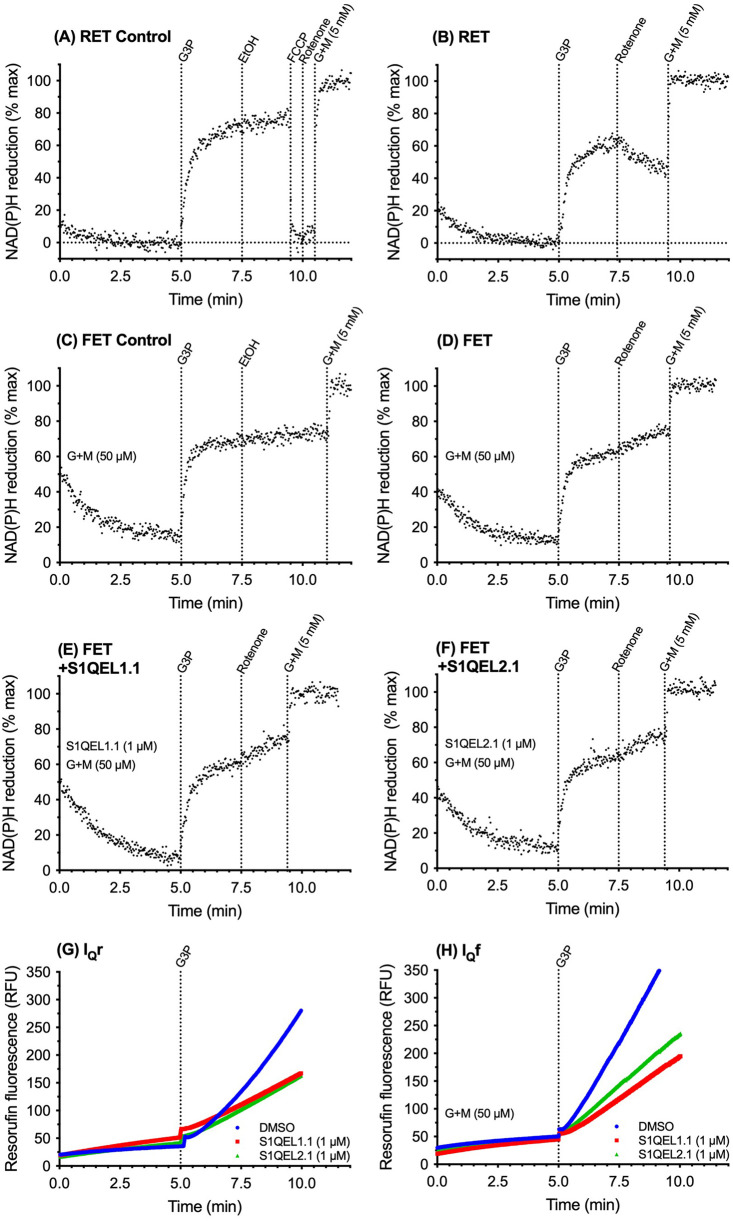
Demonstration of reverse and forward electron transport through complex I in isolated rat skeletal muscle mitochondria in cuvette-based assays assessed using the effect of rotenone challenge on NAD(P)H redox state; parallel superoxide/hydrogen peroxide production from sites I_Q_r and I_Q_f and its suppression by S1QELs. (**A**–**F**) Cuvette-based assays of NAD(P)H autofluorescence (Ex 365/Em 450). In (**A**,**B**) 0% reduction was taken as the signal after 5 min with no substrate added; in (**C**–**F**), with 50 µM glutamate plus malate (G + M) added at the start, 0% reduction was taken as the average autofluorescence signal of all runs on the same day at 5 min in the absence of added 50 µM glutamate plus malate as in (**A**,**B**). One hundred percent reduction was taken as the steady value in each trace after the addition of 5 mM glutamate plus malate. (**G**,**H**) Cuvette-based assays of superoxide/hydrogen peroxide production measured as hydrogen peroxide using the Amplex UltraRed assay. (**A**,**B**,**G**) Reverse electron transport achieved by adding 12.5 mM glycerol 3-phosphate as sole substrate at 5 min and demonstrated in (**B**) by oxidation of the matrix NAD pool on challenge with 4 µM rotenone; (**C–F**,**H)** Forward electron transport achieved by adding 50 µM glutamate plus 50 µM malate in the reaction mix and 12.5 mM glycerol 3-phosphate as additional substrate at 5 min and demonstrated in (**D–F**) by reduction of the matrix NAD pool on challenge with 4 µM rotenone. (**E–H**) 1 µM S1QEL1.1 or S1QEL2.1 as indicated added of DMSO vehicle to the reaction mix; the same volume of DMSO was added to the relevant controls. Traces are representative of duplicates each day and at least three repeats on independent mitochondrial preparations.

Figure 2 shows the FET and RET assays using endogenous matrix NAD(P)H autofluorescence, and parallel conventional Amplex UltraRed assays of superoxide/hydrogen peroxide production by isolated mitochondria in cuvettes in a fluorimeter to allow titration of substrates and exploration of conditions. Figure 2A shows the RET control. Mitochondria were added to the cuvette and endogenous NADH was allowed to oxidise and any other sources of phosphorescence and fluorescence to decay for 5 min to give a baseline of 0% NAD(P)H reduction. The addition of 12.5 mM G3P as the sole substrate then caused build-up of QH_2_ and protonmotive force, driving reverse electron flow from QH_2_ through complex I and substantial reduction of the matrix NAD pool. The addition of vehicle (ethanol) at 7.5 min had little effect. The addition of uncoupler at 9 min reoxidised the NAD pool to 0% reduced, showing that the initial 5 min relaxation time was sufficient to give essentially full oxidation of the matrix NAD pool. At 10 min rotenone and then excess 5 mM glutamate plus 5 mM malate were added to give 100% reduction in the matrix NAD pool for calibration. Figure 2B uses rotenone challenge in the same set-up to demonstrate RET when G3P alone was added as substrate. On rotenone challenge at 7.5 min the supply of electrons to the matrix NAD pool by RET from QH_2_ was prevented, so matrix NADH slowly oxidised as electrons leaked out to uncharacterised acceptors. The slow oxidation of matrix NADH on rotenone challenge is the demonstration that RET was running immediately before rotenone addition, as expected with G3P as the sole substrate.

Figure 2C,D shows conditions where FET could be demonstrated to run, validating the rotenone challenge assay. Figure 2C shows the FET control, with vehicle added at 7.5 min instead of the rotenone challenge. At zero min a small amount of G + M was added to cause a trickle of electrons into complex I and drive FET under conditions otherwise the same as those for RET in Figure 2B. Empirically we determined that 50 µM glutamate plus 50 µM malate was sufficient to drive FET in the later presence of G3P without over-reducing the NAD pool and masking the effect of the rotenone challenge. At 5 min the presence of 50 µM G + M meant that the NAD pool was still partially reduced, as determined using baselines from other runs on the same day with no NAD-linked substrate added, which were used to set the zero reduced signal. The addition of G3P at 5 min caused transient RET and further reduced the NAD pool, but this time in the steady state net electron flow was forward, driven by the extra trickle of electrons from the added G + M. This was shown by the rotenone challenge at 7.5 min in Figure 2D, which caused the NAD pool to become further reduced as the electrons trickling into the pool from G + M were no longer able to escape by FET through complex I. Vehicle addition at 7.5 min caused no further reduction (Figure 2C). Thus Figure 2D validated the assay of FET and showed that it was occurring under our conditions when G + M was added. Figure 2E,F shows that the presence of S1QEL1.1 (Figure 2E) or S1QEL2.1 (Figure 2F) did not compromise FET under the conditions of Figure 2D. They also did not affect RET under the conditions of Figure 2B, as expected from prior results [10] (not shown).

Figure 2G shows exactly parallel assays on the same mitochondrial preparation under the conditions of Figure 2A,B. The addition of G3P as the sole substrate at 5 min caused the expected large increase in superoxide/hydrogen peroxide production, known to emanate almost entirely from sites I_Q_r, G_Q_ and II_F_ [17]. These conditions drive RET, as expected and as shown by the rotenone challenge of Figure 2B. The presence of 1 µM S1QEL1.1 or 1 µM S1QEL2.1 in the assay suppressed the large site I_Q_r component of superoxide/hydrogen peroxide production under these conditions of RET as expected [5]. At this concentration, S1QELs do not affect mitochondrial bioenergetics [5].

Figure 2H introduces two crucial results of the present paper. In the conditions of Figure 2C,D (the presence of 50 µM glutamate plus 50 µM malate and 12.5 mM G3P), where FET was shown by rotenone challenge in Figure 2D to be running, the rate of superoxide/hydrogen peroxide production was similar to that measured during RET (Figure 2G). Both S1QEL1.1 and S1QEL2.1 suppressed superoxide/hydrogen peroxide production just as well under conditions of FET (Figure 2C–F,H) as they did under conditions of RET (Figure 2A,B,G), demonstrating S1QEL-sensitive superoxide/hydrogen peroxide production by site I_Q_ (site I_Q_f) during FET through complex I.

### Demonstration of reverse and forward electron transport through complex I in isolated rat skeletal muscle mitochondria and superoxide/hydrogen peroxide production from sites I_Q_r and I_Q_f using plate-reader timings

**Figure 3.**
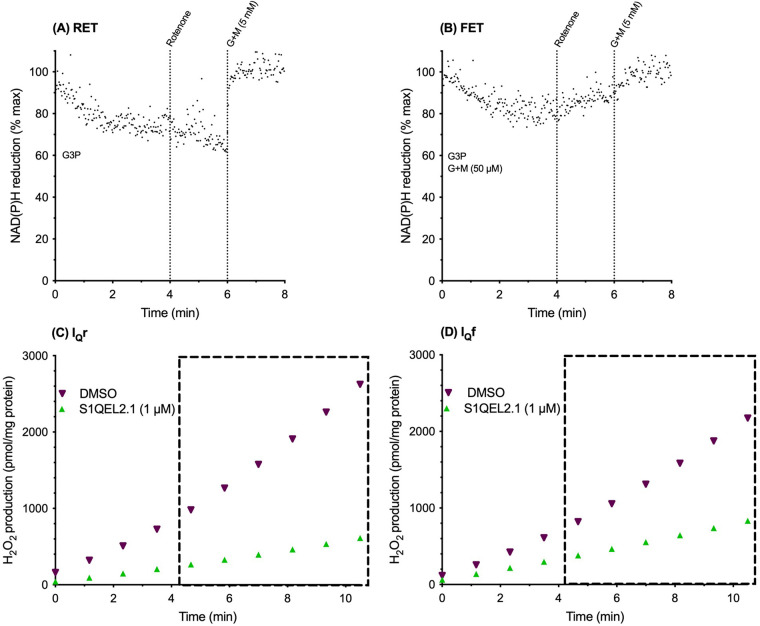
Reverse and forward electron transport through complex I in muscle mitochondria in cuvette-based assays using plate-reader timings; parallel superoxide/hydrogen peroxide production from sites I_Q_r and I_Q_f and its suppression by S1QELs in the plate-reader. (**A**,**B**) Cuvette-based assays with plate-reader timings of NAD(P)H autofluorescence, with 0% reduction taken as the average autofluorescence signal of all runs on the same day at 5 min under the conditions of Figure 2A,B and 100% reduction taken as the steady value in each trace after addition of 5 mM glutamate plus malate (G + M). (**A**) Reverse electron transport achieved by adding 12.5 mM glycerol 3-phosphate as sole substrate at *t* = 0 and demonstrated by oxidation of the matrix NAD pool upon challenge with 4 µM rotenone at 4 min; (**B**) forward electron transport achieved by adding 50 µM glutamate plus malate and 12.5 mM glycerol 3-phosphate as additional substrate at *t* = 0 and demonstrated by reduction of the matrix NAD pool upon challenge with 4 µM rotenone at 4 min. (**C**,**D**) Plate-reader assays of superoxide/hydrogen peroxide production from site I_Q_r during reverse electron transport (**C**), and from site I_Q_f during forward electron transport (**D**), measured between ∼4 min and 11 min (dotted boxes) using the Amplex UltraRed assay with DMSO or 1 µM S1QEL2.1 in DMSO added at *t* = 0. Resorufin fluorescence was calibrated using known additions of standard hydrogen peroxide; 2 µM atpenin A5 and 10 µM S3QEL3 were also added. Traces are representative of duplicates each day and at least three repeats on independent mitochondrial preparations.

Having established the principles of the rotenone-challenge assay for RET and FET in low-throughput cuvette-based assays, we transitioned to microplate assays to improve throughput. In-flight additions were less feasible in plate-reader mode, so 12.5 mM G3P and, where required, 50 µM glutamate plus 50 µM malate were added to the initial reaction mixes. To decrease irrelevant hydrogen peroxide production by other sites we also added atpenin A5 to prevent electron backflow into site II_F_ and S3QEL3 to suppress site III_Qo_. Figure 3A,B shows NAD(P)H redox state in cuvette assays using plate-reader timings with additions all at *t* = 0 min except for rotenone challenge at *t* = 4 min (and the final calibration of 100% NAD reduction at *t* = 8 min). In the same way as in Figure 2B, Figure 3A shows RET with G3P alone (NAD became less reduced on rotenone challenge). In the same way as in Figure 2D–F, Figure 3B shows FET with G3P plus 50 µM G + M (NAD became more reduced on rotenone challenge). Superoxide/hydrogen peroxide production was assessed in parallel plate-reader assays with the same substrate additions and timings. Figure 3C shows that under these conditions superoxide/hydrogen peroxide production by site I_Q_r was suppressed by S1QEL2.1 as it was in Figure 2G; Figure 3D shows that under these conditions superoxide/hydrogen peroxide production by site I_Q_f was suppressed by S1QEL2.1, as it was in Figure 2H, showing that plate-reader timings did not prevent the demonstration of RET and FET and the S1QEL-sensitivity of sites I_Q_r and I_Q_f.

### Increasing the proportion of superoxide/hydrogen peroxide originating at site I_Q_

To decrease irrelevant hydrogen peroxide production by other sites, we routinely added S3QEL3 (to suppress site III_Qo_) and atpenin A5 (to prevent electron flow back into complex II and thereby inhibit site II_F_, Figure 1) because sites III_Qo_ (4% of the total signal) and particularly II_F_ (26% of the total signal) are significant contributors during RET driven by oxidation of G3P [17,36]. Orr et al. [6] showed that S3QELs do not inhibit respiration on G3P or suppress superoxide/hydrogen peroxide production by site G_Q_. Brand et al. [5] showed that S1QELs do not suppress superoxide/hydrogen peroxide production at site G_Q_ but did not formally exclude S1QEL effects on respiration on G3P. Effects of atpenin A5 on oxidation of G3P have not been reported. Therefore, we tested whether S1QELs or atpenin A5 affected mitochondrial respiration on G3P. S1QEL1.1, S1QEL2.1 and S1QEL1.719 [25] at 1 µM, and atpenin A5 at 1–10 µM each had no significant effect on respiration of rat skeletal muscle mitochondria on 12.5 mM G3P measured in the Seahorse extracellular flux analyzer in the presence of ADP (*n* = 2–3 independent experiments each the average of three to four runs). Thus, additions of S1QELs, S3QELs and atpenin A5 do not interfere with the bioenergetics during the oxidation of G3P.

**Figure 4.**
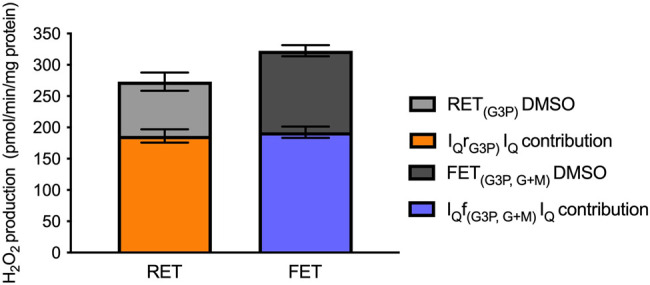
Contribution of site I_Q_ to overall superoxide/hydrogen peroxide production on glycerol-3-phosphate during reverse electron transport (site I_Q_r) and forward electron transport (site I_Q_f). Superoxide/hydrogen peroxide production by rat skeletal muscle mitochondria oxidising glycerol-3-phosphate alone (reverse electron transport (RET), site I_Q_r) or in the presence of 50 µM glutamate plus malate (forward electron transport (FET), site I_Q_f), measured in plate-reader assays in the presence of 2 µM atpenin A5 to prevent electron backflow into site II_F_ and 10 µM S3QEL3 to suppress site III_Qo_. Grey bars show overall measured rates of hydrogen peroxide production; orange and blue bars stacked within the grey bars show the rates suppressed by the addition of 1 µM S1QEL2.1. Values are means ± SEM (*n* = 3 independent experiments each the mean of at least three technical replicates).

What contribution does site I_Q_ make to hydrogen peroxide production during RET on G3P and to FET on G3P plus 50 µM G + M in the presence of S3QEL3 and atpenin A5? Figure 4 shows that the majority of superoxide/hydrogen peroxide production is from site I_Q_ in both cases. This result is expected during RET [17], but Figure 4 shows that it was also the case under FET, echoing Figures 2 and 3. The remainder is expected to be from site G_Q_ (during RET) and sites G_Q_ and I_F_ (and maybe O_F_) (during FET) [17]. The calibrated rates of superoxide/hydrogen peroxide production from site I_Q_ operating during RET as I_Q_r or during FET as I_Q_f were not different, showing that the capacity of site I_Q_ to produce superoxide/hydrogen peroxide was unchanged whether it ran during RET or FET.

### Equal sensitivity of superoxide/hydrogen peroxide production from sites I_Q_r and I_Q_f to modifiers of oxidative phosphorylation

**Figure 5.**
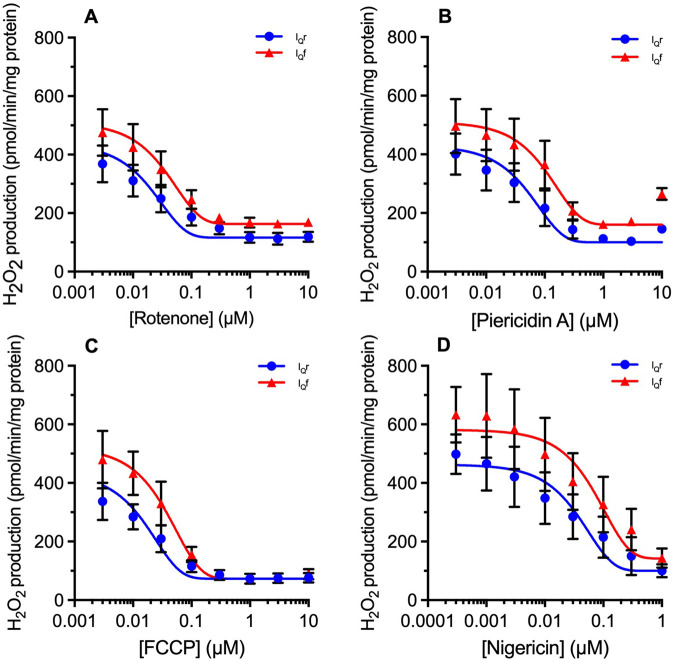
Effects of rotenone, piericidin A, FCCP and nigericin on superoxide/hydrogen peroxide production during reverse electron transport (site I_Q_r) and forward electron transport (site I_Q_f). Plate-reader assays of superoxide/hydrogen peroxide production by rat skeletal muscle mitochondria (0.2 mg/ml; 0.01 mg/well) oxidising glycerol-3-phosphate alone (reverse electron transport, site I_Q_r) or in the presence of 50 µM glutamate plus malate (forward electron transport, site I_Q_f), in the presence of 2 µM atpenin A5 to prevent electron backflow into site II_F_ and 10 µM S3QEL3 to suppress site III_Qo_. Effects of (**A**) rotenone; (**B**) Piericidin A; (**C**) FCCP; (**D**) Nigericin, each added at the start of the experiment. Residual rates were not subtracted because they presumably include contributions from sites G_Q_ and I_F_ (and, with glutamate plus malate added, site O_F_) that alter during the titrations. Curves were fit using the non-linear curve function (variable slope; four parameter logistic) of Prism 7. Values are means ± SEM (*n* = 3 independent experiments each the mean of 3 technical replicates).

Next, we explored the characteristics of site I_Q_f using the new FET conditions and the I_Q_f assay to see if it behaved like site I_Q_r. Superoxide/hydrogen peroxide production at site I_Q_r is known to be sensitive to the classic Q-site electron transport inhibitors rotenone and piericidin A, which act strongly as inhibitors to prevent RET into the site [7–9,18] and also weakly as S1QELs [10]. FCCP acts as a protonophore to dissipate protonmotive force, decreasing both the driving force for RET into the Q-site and also the high protonmotive force needed to support site I_Q_r activity [12,18]. Nigericin, under our conditions of high extramitochondrial K^+^ concentration, dissipates the ΔpH needed to support the catalytic activity of site I_Q_r while increasing mitochondrial membrane potential and maintaining protonmotive force [12]. Figure 5 explores whether site I_Q_ operating in I_Q_f mode retains sensitivity to these reagents.

Comparison of superoxide/hydrogen peroxide production from sites I_Q_f and I_Q_r shows that the two modes of site I_Q_ have generally very similar properties. The uninhibited rates attained under our standard conditions of high protonmotive force, high ΔpH and high QH_2_/Q ratio were very similar (Figures 2–5). They were both equally sensitive to inhibition by complex I Q-site inhibitors (rotenone and piericidin A, Figure 5A,B), and to decreasing protonmotive force and QH_2_/Q ratio (FCCP, Figure 5C) or decreasing ΔpH at presumed constant protonmotive force (nigericin, Figure 5D). Inhibition of superoxide/hydrogen peroxide production at site I_Q_ during FET (i.e. site I_Q_f) by the complex I Q-site inhibitors confirms our previous conclusion [13] that site I_Q_ is distinct from site I_F_, which is not inhibited but enhanced in the presence of these inhibitors. Distinct sites I_Q_ and I_F_ remains the simplest and most obvious interpretation of all the data from our and other laboratories; however, it remains formally possible that inhibition of the Q-site by rotenone or piericidin A alters the conformation of the distant flavin site in some particular way, and ‘site I_Q_' is really the flavin in a different state, or the inverse — that ‘site I_F_' is really the Q-site with complex conformational constraints.

In previous work exploring site I_Q_f, Lambert et al. [12,18] found that superoxide/hydrogen peroxide production from site I_Q_f could be measured under conditions of high protonmotive force, high pH, and high QH_2_/Q ratio, but only in the presence of high concentrations of complex I electron transport inhibitors such as piericidin A, rotenone or myxothiazol. At first sight that is the opposite of what we report in Figure 5, where Q-site electron transport inhibitors suppress superoxide/hydrogen peroxide production not only from site I_Q_r, but also from site I_Q_f. This contradiction can possibly be resolved by the observation that at the highest concentrations of piericidin A (10 µM in Figure 5B) I_Q_f activity reappeared, perhaps mimicking the conditions of Lambert et al. [18].

### Equal sensitivity of superoxide/hydrogen peroxide production from sites I_Q_r and I_Q_f to S1QELs

**Figure 6.**
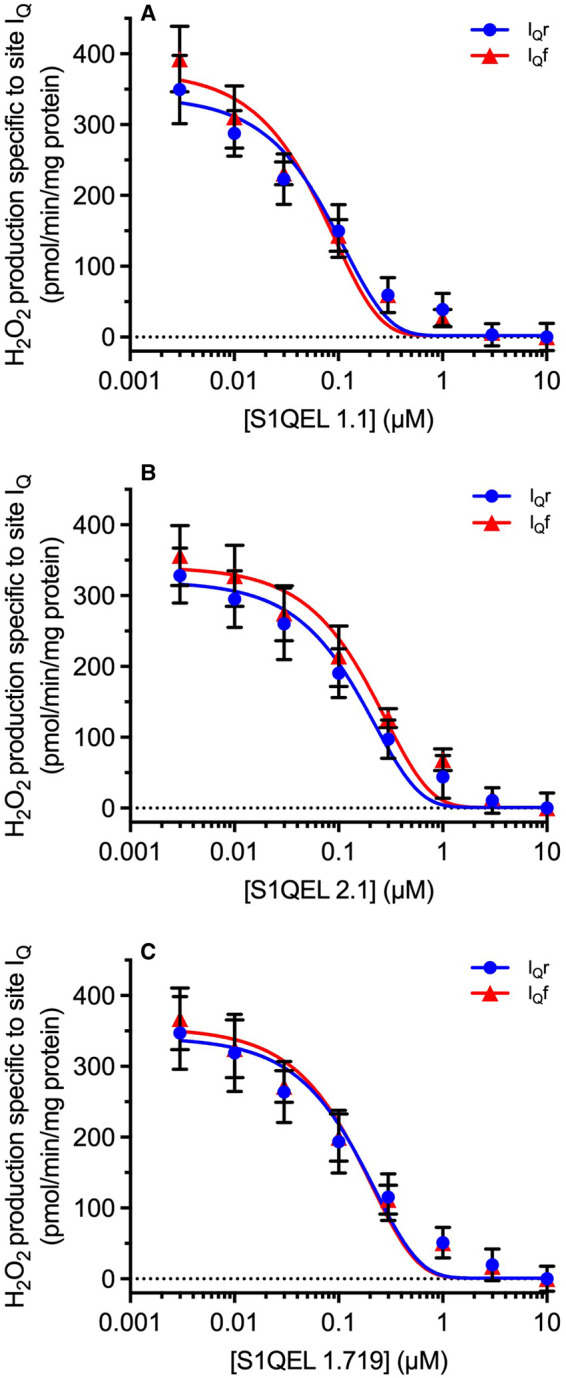
Effects of S1QELs on superoxide/hydrogen peroxide production during reverse electron transport (site I_Q_r) and forward electron transport (site I_Q_f). Plate-reader assays of superoxide/hydrogen peroxide production by rat skeletal muscle mitochondria (0.2 mg/ml; 0.01 mg/well) oxidising glycerol-3-phosphate alone (reverse electron transport, site I_Q_r) or in the presence of 50 µM glutamate plus malate (forward electron transport, site I_Q_f), in the presence of 2 µM atpenin A5 to prevent electron backflow into site II_F_ and 10 µM S3QEL3 to suppress site III_Qo_. Effects of (**A**), S1QEL1.1 (Nominal IC_50_ values I_Q_r, 70.3 ± 2.2 nM; I_Q_f, 64.0 ± 9.3 nM); (**B**) S1QEL2.1 (Nominal IC_50_ values I_Q_r, 146.3 ± 29.0 nM; I_Q_f, 172.7 ± 10.3 nM); (**C**) S1QEL1.719 (Nominal IC_50_ values I_Q_r, 125.3 ± 6.2 nM; I_Q_f, 120.7 ± 3.5 nM), each added at the start of the experiment. These IC_50_ values are labelled ‘nominal’ because they were based on added, not measured, concentrations of S1QELs. The small residual rate with saturating S1QEL presumably included contributions from sites G_Q_ and I_F_ (and, with glutamate plus malate added, site O_F_) that did not change during the titrations, so this rate was subtracted in each panel, leaving only the signal specific to site I_Q_, and allowing determination of IC_50_ values. Curves were fit using the non-linear curve function (variable slope; four parameter logistic) of Prism 7. Values are means ± SEM (*n* = 3 independent experiments each the mean of three technical replicates).

Figures 2–4 show that the rates of superoxide/hydrogen peroxide production by sites I_Q_r and I_Q_f are similar, and that they can each be suppressed by high concentrations of S1QEL1.1 or S1QEL2.1. Figure 6 investigates whether the rates of superoxide/hydrogen peroxide production by sites I_Q_r and I_Q_f are equally sensitive to S1QELs. Figure 6A–C shows that they are: the titrations of sites I_Q_r and I_Q_f with three different S1QELs each overlay very closely, and the nominal IC_50_ values for suppression of site I_Q_r and I_Q_f were indistinguishable. We conclude that superoxide/hydrogen peroxide production by site I_Q_ is equally sensitive to S1QELs whether it operates during RET as site I_Q_r, or during FET as site I_Q_f.

### No confounding effects of heterogeneity of the mitochondrial preparations

Can the conclusions of active superoxide/hydrogen peroxide production by site I_Q_f during FET and S1QEL suppression of I_Q_f be in error, because of a hypothetical possibility that there exists a subset of mitochondria running I_Q_r even under conditions of nominal I_Q_f and bulk rotenone-challenge reduction of NAD? Perhaps, because of natural heterogeneity or isolation artefacts, a significant proportion (say 40%) of the mitochondria in our preparations continues to run RET and site I_Q_r (and responds to rotenone challenge by oxidising its NADH pool) when the remaining 60% run FET and respond to rotenone challenge by reducing their NAD pool but produce little superoxide/hydrogen peroxide. In this scenario, these 40% of the mitochondria run site I_Q_r and contribute the entire rate of superoxide/hydrogen peroxide production we assign to site I_Q_f, yet contribute insufficient NADH signal on rotenone challenge to dominate the bulk NAD reduction seen on rotenone challenge.

**Figure 7.**
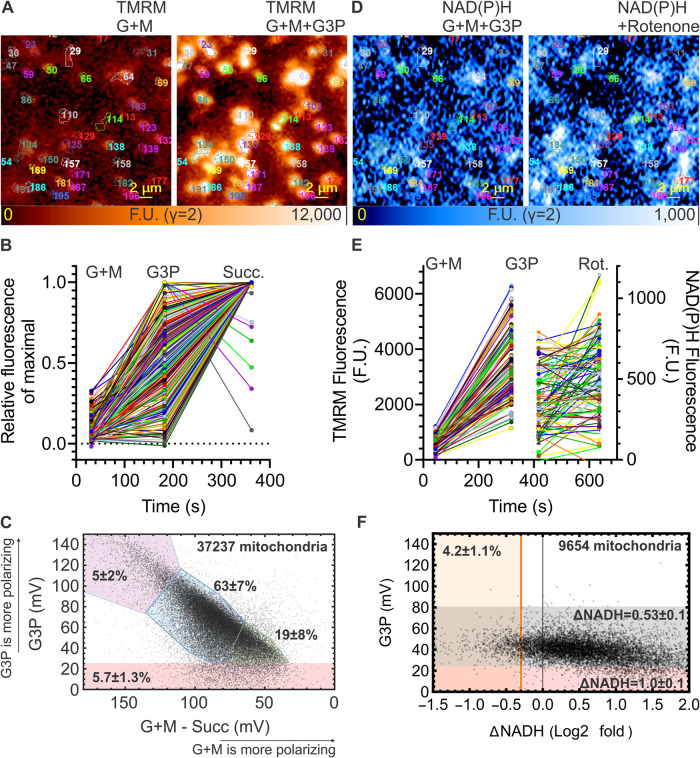
Heterogeneous substrate oxidation in rat skeletal muscle mitochondria. (**A**) Wide-field fluorescence micrographs of TMRM in isolated rat skeletal muscle mitochondria immobilised on glass in the presence of the indicated substrates. The two cropped frames show the same mitochondria before and after glycerol 3-phosphate addition with individual mitochondria marked by automated morphological segmentation. (**B**) Fluorescence intensity time courses of TMRM as in (**A**), recorded over individual mitochondria during the sequential addition of the indicated substrates. Data were normalised between the frame background and the maximal fluorescence for the entire time course for each mitochondrion. (**C**) Scatter plot analysis of ΔψM responses of individual mitochondria to glycerol 3-phosphate (when added after glutamate plus malate (G + M)) as a function of polarisation by glutamate plus malate alone. The polarisation by glutamate plus malate was referenced to the ΔψM provided by succinate at the end of the experiment (see (**B**)), therefore, smaller values (difference) indicate a larger initial polarisation by glutamate plus malate, assuming that succinate was maximally polarising. Coloured regions indicate arbitrarily chosen classes of mitochondria with differing extent of response to the two substrates. Mean ± SE values describe the size of these classes for experimental replicates (*n* = 4). (**D**) Wide-field fluorescence micrographs of NAD(P)H autofluorescence recorded in the same mitochondria as in (**A**) following the TMRM frames, before and after the addition of rotenone. (**E**) Fluorescence intensity time courses of TMRM, followed by recording of NAD(P)H autofluorescence over the same mitochondria while substrates or rotenone were added as indicated. (**F**) Scatter plot analysis of ΔψM responses of individual mitochondria to glycerol 3-phosphate (added after 50 µM glutamate plus malate) as a function of response to rotenone in the presence of glutamate plus malate + glycerol 3-phosphate. Mean ± SE values describe experimental replicates (*n* = 4). Note that only 4.2% of mitochondria responded to rotenone by significant oxidation of NAD(P)H (orange line defined at log_2_ of 1–2 times the average SE of the repeated autofluorescence determinations after rotenone addition), and the 1% of mitochondria that did not polarise well with glycerol 3-phosphate responded to rotenone with a stronger increase in autofluorescence (pink 1.0 ± 0.1 vs grey 0.53 ± 0.1 log_2_-fold values, *P* < 0.01, *n* = 4). G + M, 50 µM glutamate plus 50 µM malate; G3P, 12.5 mM glycerol 3-phosphate; succ, 5 mM succinate.

To test this possibility, we measured the heterogeneity of our mitochondrial preparation using fluorescence microscopy of single mitochondria stained with tetramethylrhodamine methyl ester (TMRM), a probe of the mitochondrial membrane potential (ΔψM), combined with imaging of NAD(P)H autofluorescence (Figure 7). Specifically, we quantified the ‘RET and I_Q_r population', defined as the fraction of the mitochondrial population in the presence of 50 µM G + M that polarised strongly on the addition of G3P (to favour any RET), and also responded to rotenone challenge with NADH oxidation (rather than reduction), showing that this sub-population was running RET (and site I_Q_r), not FET (and site I_Q_f) as inferred from the bulk results.

First, we compared the magnitudes of ΔψM provided by G3P and by G + M. Because the size, and therefore the fluorescence signal, of individual mitochondria varies, this first set of experiments, which measured single-mitochondrion TMRM intensities only, was finished by the addition of succinate as a calibration of maximal ΔψM (Figure 7B,C). This approach enabled us to calculate the mV hyperpolarisation in individual mitochondria upon the addition of G3P in the presence of G + M (Figure 7C, *y*-axis). Because a precise zero mV calibration point was not feasible, the polarisation supported by G + M alone was measured indirectly by expressing how much less polarised the individual mitochondria were in the presence of G + M than with G3P and succinate also added (Figure 7C, *x*-axis). Thus, a smaller magnitude (difference) on this axis means stronger polarisation by G + M alone. Overall, the magnitude of the response of individual rat skeletal isolated mitochondria to G3P in the presence of G + M varied as a continuum from no response to strong response, with no clearly distinct subpopulations. With an arbitrary definition of categories, Figure 7C indicates that most mitochondria (63 ± 7%; *n* = 4; blue) showed a medium increase in polarisation for each of the consecutive additions of G + M and G3P. A weaker G3P and stronger G + M signal was observed in 19 ± 8% of mitochondria (green); such a population may run mostly FET and make a disproportionate contribution to the rotenone-evoked increase in NAD reduction. Importantly, the potential ‘RET and I_Q_r population', giving a strong G3P response after very weak polarisation by G + M, was rare (5 ± 2%, purple). A small population (5.7 ± 1.3%, pink) gave little response to G3P. Thus, only ∼5% of the mitochondrial population gave a large increase in ΔψM when provided with G3P in the presence of 50 µM G + M, suggesting they might still be running RET even when the bulk population ran FET.

Next, we measured the polarisation by G3P when added in the presence of G + M as in Figure 7A, then instead of calibrating with succinate as in Figure 7B,C, we tested the effect of FCCP or rotenone on NAD(P)H autofluorescence in the same mitochondria. As a positive control (not shown), we first challenged with FCCP (4 µM); 85 ± 2% of mitochondria responded with a significant oxidation of NADH, showing the sensitivity of the method. In the main experiment, when challenged by the addition of rotenone (Figure 7D–E) most of the mitochondria showed an increase in NAD(P)H autofluorescence, as expected from Figures 2D and 3B, consistent with FET. However, a small fraction showed a decrease, consistent with RET. Figure 7F quantifies the relationship between ΔNADH and the polarisation by G3P. 65% of the mitochondria responded to rotenone addition by significantly increasing the reduction of their NADH/NAD^+^ pool, showing they were running FET under our experimental conditions. Only 4.2 ± 1.1% (*n* = 4 different runs), mostly the potential ‘RET and I_Q_r population' that were more strongly polarised by the addition of G3P in the presence of G + M, responded to rotenone challenge by a significant oxidation of NADH to NAD^+^, showing they were indeed running RET and presumably site I_Q_r.

Since the rates of superoxide/hydrogen peroxide production by sites I_Q_r and I_Q_f were indistinguishable (Figures 4–6), the 4.2% ‘RET and I_Q_r population' population could only explain the results presented in this paper if it was essentially the only population producing superoxide/hydrogen peroxide under all our conditions. This possibility seems unlikely given that the other 95% were well-polarised in the presence of G + M and G3P, so are expected to have been the major source of superoxide/hydrogen peroxide under our standard I_Q_r conditions. Thus, only ∼4% of the heterogenous mitochondrial population was running RET under our conditions of overall FET, and this 4% ‘RET and I_Q_r population' was very unlikely to be the only population producing superoxide/hydrogen peroxide under all our experimental conditions of reverse and FET, strongly supporting our conclusion that site I_Q_f was correctly assigned in the preceding figures. Notably, 30 ± 9% of mitochondria did not change NAD(P)H fluorescence significantly in response to the rotenone challenge, indicating a fairly large population with close-to-stalled electron transport. It is possible that superoxide/hydrogen peroxide production from site I_Q_ occurs when FET slows or stalls due to high protonmotive force, ΔpH and QH_2_/Q ratio. In this scenario 30% of complex I is in (near) equilibrium, site I_Q_ is active, and challenge with rotenone causes no significant change in NAD(P)H.

We conclude that site I_Q_, running FET (65%) or near equilibrium (30%), but not running RET (4%), is the source of superoxide/hydrogen peroxide in mitochondria under our contrived conditions of FET in isolated mitochondria, and that heterogeneity of our mitochondrial preparation does not lead to erroneous identification of site I_Q_f in the bulk preparation driven by site I_Q_r in a smaller subset.

### Site I_Q_f runs in cells

The experiments in Figures 2–7 show that in isolated mitochondria, site I_Q_ running during FET (site I_Q_f) produces superoxide/hydrogen peroxide at high rates and is fully S1QEL-sensitive. Is the same true of mitochondria in intact cells? From theoretical considerations we expect FET to be the default in cells, generating ATP from the oxidation of NADH using all three mitochondrial proton-pumping complexes, I, III and IV [32,40], whereas FET using only complexes III and IV to drive ATP synthesis while electrons flow in RET from QH_2_ to NAD is expected to occur only under unusual or contrived conditions, such as the transient oxidation of excess succinate during ischaemia-reperfusion [27,28]. There is an expectation of FET in bulk cell cultures and tissues, as these generally respond to rotenone challenge by a reduction of their matrix NAD(P) pool [41–44]. Therefore, the observed suppression of hydrogen peroxide by S1QELs in a variety of intact cell types [20–23] is most likely to be driven by site I_Q_f rather than site I_Q_r. Nonetheless, cell populations may be heterogeneous, and, in theory, the observed bulk superoxide/hydrogen peroxide production might originate from a sub-population of cells with unusual characteristics. Note that at 1–2 µM S1QELs do not affect cellular bioenergetics [5].

**Figure 8.**
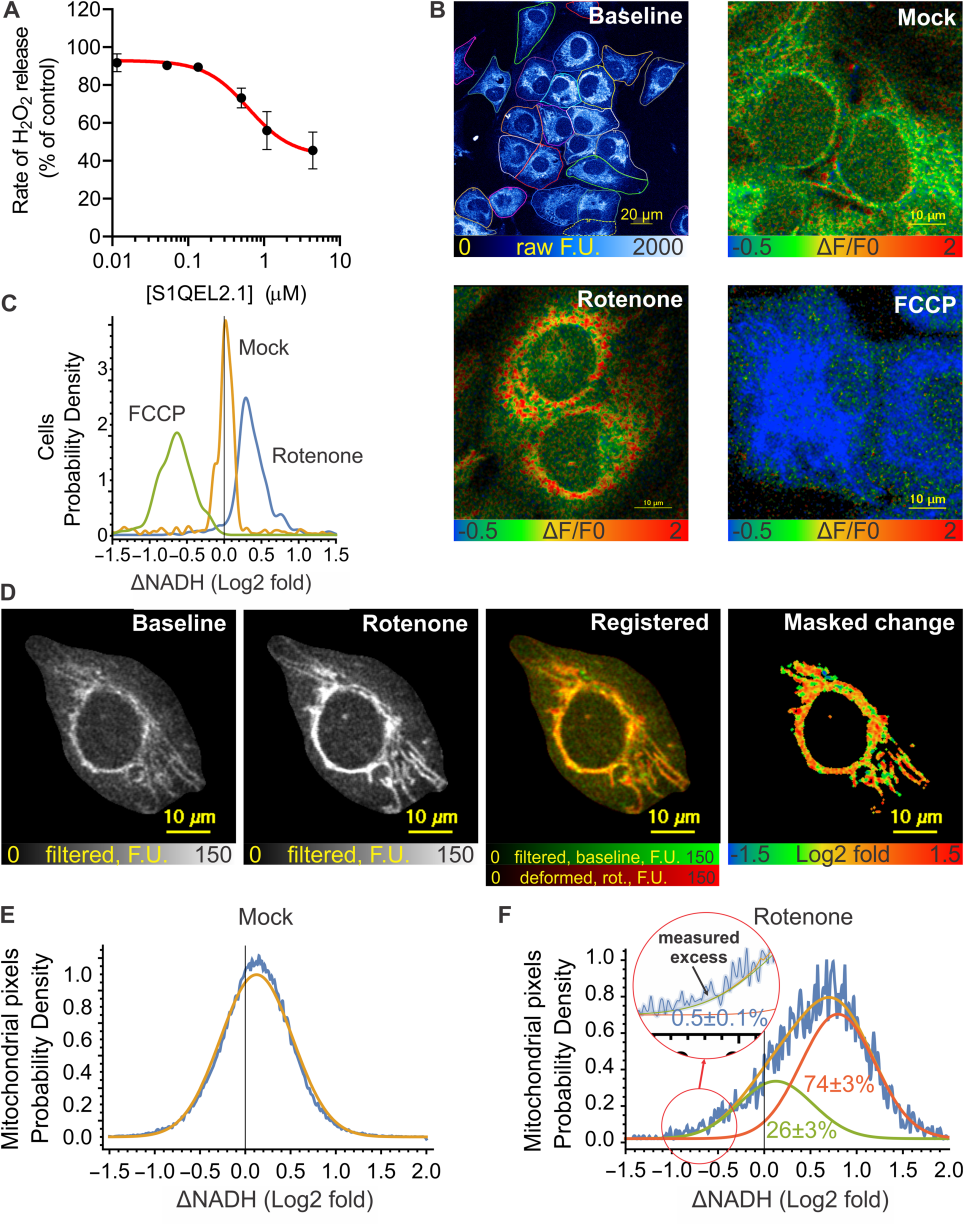
Hydrogen peroxide release and NAD(P)H redox state in Fa2N-4 cells. (**A**) Effect of S1QEL2.1 on hydrogen peroxide release from Fa2N-4 immortalised hepatocytes determined by the Amplex UltraRed/HRP assay in the medium above the cell monolayer. (**B**) NAD(P)H autofluorescence of Fa2N-4 cells. From top left, representative full-frame two-photon micrograph of NAD(P)H autofluorescence in basal conditions with single Fa2N-4 cells outlined using Cellpose2, and magnified regions showing the effects of vehicle (mock), rotenone (4 µM), or FCCP (4 µM) in pseudocolour coding after pixelwise Δ*F*/*F*_0_ calculation. (**C**) Distribution of fluorescence changes in single whole cells following the indicated treatment. Δ*F*/*F*_0_ values were calculated per cell; the histogram shows pooled cell populations from three experiments. Virtually all cells responded to rotenone by increased autofluorescence (NAD(P) reduction), and to FCCP by decreased autofluorescence (NAD(P)H oxidation). (**D**) Cropped and processed two-photon micrographs of NAD(P)H autofluorescence illustrating the analysis of NAD(P)H responses to rotenone within the mitochondrial population in individual cells. Representative of 185 cells from three experiments. From left; frames at baseline and 1 min after rotenone were intensity rescaled and Wiener filter smoothed (greyscale) then subjected to deformable image registration (baseline, green; deformed rotenone frame, red). Log_2_-fold changes were calculated pixelwise and images were masked for mitochondria (visible before and after rotenone; mask is black, valid pixels are in pseudocolour). (**E**) Distribution of log_2_-fold changes in pixel intensities over mitochondria in a pooled vehicle-treated cell population. The orange Gaussian fit line indicates the spread of measured intensity changes due to noise, movement or fluctuations of redox state. (**F**) Distribution of log_2_-fold changes in pixel intensities over mitochondria in a representative (of 185) rotenone-treated cell. The pixel value histogram (blue trace) was fitted with the sum (orange) of two normal distributions comprising rotenone-unresponsive (green) and rotenone-responsive (red) pixel populations. The inset indicates how the fraction of mitochondria undergoing rotenone-induced oxidation was measured as an excess pixel count above the orange fit line, at significantly negative log_2_-fold changes (defined as 2*σ* of the fit on mock-treated cells). Mean ± SE values describe mitochondrial subpopulations (as the area under or between the curve(s)) for experimental replicates (*n* = 3), where values were calculated for single cells and then pooled for each experiment. F.U., fluorescence units.

Figure 8A–C reports bulk superoxide/hydrogen peroxide production by site I_Q_ in Fa2N-4 immortalised hepatocytes, and the evidence that this occurs during bulk FET in the mitochondria (i.e. site I_Q_f) within the cells. In these cells, based on the maximal suppression achieved by S1QEL2.1, site I_Q_ was responsible for about half of total cellular hydrogen peroxide production (Figure 8A), making them well-suited for more stringent evaluation. To examine the effects of the rotenone challenge, NAD(P)H autofluorescence was recorded at high resolution using multiphoton microscopy allowing analysis of many cells per view field and also of individual mitochondria within cells. To minimise (photo)toxic effects no mitochondrial markers were used, and only single images before and after treatment were recorded. Rotenone and FCCP-induced changes in autofluorescence are expected to reflect changes in mitochondrial NADH and these reagents are expected to have little effect on cytosolic NADH/NAD^+^ or NADPH/NADP^+^ pools. All cells responded to FCCP with a decrease in autofluorescence (Figure 8B,C), showing that mitochondrial NADH was sufficiently reduced at baseline so its oxidation could be easily detected. Virtually all cells (97 ± 4%; *n* = 4) responded to rotenone challenge with an increase in autofluorescence (Figure 8B,C), mirroring the mitochondrial results in Figures 2 and 3. This response to rotenone challenge recapitulates the literature cellular responses cited above, and indicates that FET occurs in the bulk population and in individual cells under basal conditions matching those of the hydrogen peroxide assay (Figure 8A).

### No confounding effects of heterogeneity of mitochondria in cells

Next, we assessed the direction of electron flow on the subcellular scale to test if RET was present in a fraction of mitochondria within single cells. To this end, Figure 8D–F analyses the heterogeneity of the response of NAD(P)H autofluorescence to rotenone challenge to test if RET was present in a fraction of cells or in a fraction of mitochondria within single cells. Individual mitochondria were visible as punctate NAD(P)H two-photon autofluorescence in cells in the basal condition, and after rotenone and mock treatments (Figure 8B,D). To quantify tentative subpopulations of mitochondria with differing responses to rotenone, log_2_-fold changes of pixel intensities were calculated between the baseline and rotenone frames. Mitochondria were highly motile; therefore, the post-treatment frame was warped using deforming image registration for the best match to the baseline frame. We expected that any mitochondria running reverse electron flow would become oxidised, as observed for the bulk with FCCP. Whether this happens was tested by analysing the distribution of log_2_-fold changes of pixel intensities between before- and after-treatment frames. There was a narrow, symmetric normal distribution of pixel intensity changes in response to mock treatment, which we attribute to the effects of motility and noise (Figure 8E). In rotenone-treated cells, this distribution widened and shifted to the right (Figure 8F), indicating an increase in fluorescence in most areas, and a heterogeneous response of mitochondria to rotenone. We then asked if the left tail of the distribution can be explained by the presence of mitochondria not responding to rotenone or oxidising on rotenone challenge. We accounted for a similar statistical spread of the intensity changes over time as observed in the mock treatment by fitting the distributions measured in the rotenone-treated cells with a sum of two normal distributions, a non-responder and an arbitrary magnitude responder population. 26 ± 3% of mitochondria (*n* = 3 experiments, where a total of 185 cells were individually analysed) were estimated to respond poorly or not at all to rotenone, while 74 ± 3% responded with a 50 ± 10% mean increase in fluorescence intensity. The population of mitochondria oxidising on rotenone challenge was measured by the excess of measured pixel counts over the left tail of the distribution of the log_2_-fold values (Figure 8F inset). This population was very small, 0.5 ± 0.1% of cellular mitochondria. As a control, we simulated RET by masking 6.4% of mitochondria in the rotenone frame with background intensity values in the raw recordings. Based on the FCCP treatments such change in fluorescence is expected if NADH goes fully oxidised upon rotenone treatment. This was robustly detected by the excess pixel count method described above as 5% of mitochondria becoming oxidised (data not shown).

Overall, Figure 8 shows that on average almost all Fa2N-4 cells (97%) ran FET through complex I. However, the cells contained a significant population (26%) of mitochondria with complex I close to equilibrium (or stalled), and a very small population (0.5%) of mitochondria running RET. Considering the similar rates of superoxide/hydrogen peroxide production by sites I_Q_f and I_Q_r (Figures 4 and 5), it is unlikely that the 0.5% of mitochondria that exhibit RET in cells support the whole of the observed large S1QEL-sensitive superoxide/hydrogen peroxide production (Figure 8A), so we conclude that in cells, as in mitochondria, site I_Q_ of complex I generates superoxide/hydrogen peroxide at high rates that are sensitive to S1QELs even when it runs during FET as site I_Q_f. Given the relatively high abundance of mitochondria operating close to the equilibrium of complex I in these cells (26%), it may be best to consider that site I_Q_ is a potent generator of superoxide/hydrogen peroxide in mitochondria and in cells regardless of the net direction of electron flow, and that all modes are equally S1QEL-sensitive. The conventional assay of superoxide/hydrogen peroxide production by site I_Q_ as site I_Q_r during RET in isolated mitochondria [2,3,11,45] is an experimental convenience, not a physiological necessity.

## Discussion

Mitochondrial complex I in mitochondria and cells often runs close to equilibrium, with the redox potential drop for electrons flowing from the matrix NADH pool to the membrane Q pool (2*Δ*E*_h_) closely matched by the resulting protonmotive force set up by proton pumping at complex I or the other electron transport chain complexes (4*pmf) [32]. For this reason, small changes in conditions, such as increased electron flow into the NAD pool from upstream dehydrogenases, can further reduce the NAD pool, thereby increasing 2*Δ*E*_h_, and causing complex I to run FET until conditions re-equilibrate. Conversely, increased electron flow into the Q pool from complex II or mitochondrial G3P dehydrogenase can further reduce the Q pool, thereby decreasing 2*Δ*E*_h_, or a decrease in ATP demand by the cell can raise 4*pmf, with either effect causing complex I to run RET until conditions re-equilibrate. In this paper, we introduce a simple assay to determine if electron flow through mitochondrial complex I is in the thermodynamically favourable forward or thermodynamically unfavourable reverse direction in any particular situation: on blocking electron flow through the Q-site of complex I, the endogenous matrix NAD pool will become more reduced if the flow prior to the challenge was forward, but more oxidised if the flow was reverse. We use this assay to show in the model system of isolated rat skeletal muscle mitochondria that superoxide/hydrogen peroxide production by complex I, with the diagnostic characteristics of site I_Q_ (dependence on high QH_2_/Q ratio, high proton motive force and, independently, high ΔpH) can be equally great whether RET or FET is occurring. Importantly, the activity of site I_Q_r can now be demonstrated without the need for the addition of high concentrations of complex I inhibitors as originally reported [18]. We then show that both modes of site I_Q_ (I_Q_r and I_Q_f) are equally sensitive to specific suppressors of superoxide/hydrogen peroxide production by site I_Q_, S1QELs. The observation that two quite different S1QEL chemotypes (S1QEL1 series and S1QEL2 series [5]) are equally effective lends confidence to the conclusion that the S1QEL effects are on-target. I_Q_r and I_Q_f are also equally sensitive to rotenone and piericidin A, inhibitors that block the Q-site of complex I. Finally, we show that superoxide/hydrogen peroxide production by site I_Q_ in cells occurs during FET and is S1QEL-sensitive.

These observations resolve the intriguing and important question raised in the Introduction: S1QELs have potent effects in cells [5,20–24] and *in vivo* [25,26], yet *in vitro* under physiologically plausible conditions they had only been shown to suppress superoxide/hydrogen peroxide production during RET [5,45]. Our new results show that S1QELs are equally effective during FET and RET, so the effectiveness of S1QELs in cells and *in vivo* does not mean that RET (and superoxide/hydrogen peroxide production by site I_Q_r) is common in cells and *in vivo* under normal and pathological conditions. Instead, it means that site I_Q_ can run during forward electron flow as site I_Q_f, yet can still be fully S1QEL-sensitive, and the effects S1QELs in cells and *in vivo* do not depend on the direction of electron transport, whether it is forward or reverse (or, probably, stalled near the equilibrium point of complex I). The direction of electron flow cannot be inferred from the effects of S1QELs or complex I inhibitors such as rotenone on superoxide/hydrogen peroxide production, as is sometimes done, e.g. [29], but instead should be confirmed independently, for example using the rotenone challenge assay introduced in the present paper. Arguments that RET *per se* (rather than conditions of high QH_2_ reduction, protonmotive force and ΔpH [8,12,13]) has special properties with regard to mitochondrial superoxide/hydrogen peroxide production [29–31] are likely premature.

## Materials and methods

### Materials

S1QEL1.1, S1QEL2.1 [5], S1QEL1.719 [25] and S3QEL3 [6] were provided by Calico Life Sciences LLC (South San Francisco, CA) and AbbVie Inc. (Chicago, IL). They were maintained as 10 mM stocks in dimethylsulfoxide at room temperature, shielded from bright light and diluted in dimethylsulfoxide as required before use. Amplex UltraRed (Cat No. A36006) was from ThermoFisher; atpenin A5 (Cat No. 11898) and FCCP (Cat No. 15218) from Cayman Chemicals; piericidin A (Cat No. 2738-64-9) from Santa Cruz Biotechnology; and horseradish peroxidase (HRP) (Cat No. P8125), superoxide dismutase 1 (SOD1) (Cat No. S7571), rotenone (Cat No. R8875), myxothiazol (Cat No. T5580), succinate (Cat No. 14160), glycerol 3-phosphate (Cat No. 94124), malonate (Cat No. 792535) and glutamate (Cat No. 1294976) were from Sigma.

### Rat skeletal muscle mitochondria

All animal experiments took place in the Buck Institute for Research on Aging. Scientific ethics and the animal protocol were approved by the Institutional Animal Care and Use Committee of the Buck Institute (License/protocol No. 10241) in accordance with the National Institutes of Health guide for the care and use of Laboratory animals (NIH Publications No. 8023, revised 1978). Rats were euthanised by CO_2_ asphyxiation followed by bilateral thoracotomy. Mitochondria were isolated from rat hindlimbs in Chappell–Perry buffer (CP-1; 100 mM KCl, 50 mM Tris HCl, 2 mM EGTA, pH 7.4) at 4°C by standard procedures [46].

### Superoxide/hydrogen peroxide production and reverse or forward electron transport in isolated mitochondria

Cuvette- and plate-based assays of superoxide/hydrogen peroxide production during RET using 12.5 mM G3P (or during FET using 12.5 mM G3P, 50 µM glutamate plus 50 µM malate) as substrate were conducted at 37°C as described [45] using 0.2 mg of mitochondrial protein/ml in 3 ml (plastic cuvettes, Varian Cary Eclipse spectrofluorometer (Ex 560 nm/Em 590 nm)) or 0.2 mg mitochondrial protein/ml, 0.01 mg/well (Pherastar FS (BMG) fluorescence microplate reader, Ex 540 nm/Em 590 nm), in KHEB medium (120 mM KCl, 3 mM HEPES, 1 mM EGTA, 0.3% w/v bovine serum albumin, pH 7.4) containing 25 U/ml SOD1, 25 µM Amplex Ultrared, and 5 U/ml HRP, plus dimethyl sulfoxide (DMSO) or treatments as indicated. Note that about half of the hydrogen peroxide produced in the mitochondrial matrix is consumed by NADH-dependent antioxidant systems before it is released and measured by this assay [47]. In the present paper, no correction was made for this effect, resulting in a significant underestimate of the absolute rates of hydrogen peroxide release in Figures 3–6. The ∼10% difference in NADH reduction level during the assay of I_Q_r and I_Q_f (Figure 3) will have changed antioxidant activity slightly, but unlikely enough to significantly affect our conclusions. In parallel experiments lacking Ample Ultrared RET or FET was determined by the effects of 4 µM rotenone challenge on NAD(P)H autofluorescence followed in the fluorimeter at Ex 365 nm/Em 450 nm.

### Respirometry of isolated mitochondria

Mitochondrial respiration was assayed at 37°C using an Agilent Seahorse XFe-24 as described previously [46], using 12.5 mM G3P as substrate.

### Determination of ΔψM and NAD(P)H autofluorescence in single isolated mitochondria

Mitochondria were immobilised in coverglass-bottomed 96-well microplates (Greiner Sensoplate) by centrifugation for 15 min at 1000 ***g*** in KHEB medium at 4°C. The microplate was warmed in the 37°C heated environment chamber of a Nikon Eclipse Ti-PFS fully motorised wide-field fluorescence microscope equipped with a Lambda 821 LED light source with Lambda 10-3 emission filter wheel and Andor iXon Life 888 EMCCD camera, controlled by NIS Elements 5.20. TMRM (100 nM) was added to the wells, and in successive additions substrates were added without changing the TMRM concentration. TMRM was imaged using a Super Fluor 40× NA1.3 oil lens, a 586/20 nm excitation filter on a 560 nm LED, a 593 nm dichroic mirror, and a 641/75 nm emission filter capturing the centre 512 × 512 pixels area of the camera with no binning and with 1.5× optical zoom (0.17 µm/pixel resolution). Multiple (typically 25) view fields were recorded in one to three wells for 3 time points per substrate condition. In some experiments, TMRM imaging was followed by recording frames of NAD(P)H autofluorescence in the same view fields using a 340 nm LED, 414 nm dichroic mirror and 460/80 nm emission filter. The first autofluorescence frame matched the substrate condition of the last TMRM frame and then the recording of three more frames followed the application of rotenone (4 µM) or FCCP (4 µM) by 1 min delay. Recordings were analysed in Image Analyst MKII 4.2.1 (Image Analyst Software, Novato, CA) by performing image registration, background subtraction and ROI-based intensity measurement. Background was subtracted first as a blank image and then frame-by-frame using the ‘Mean of pixels below percentile of max projection (dilated mask)' method. Single-mitochondria ROIs were generated by morphological segmentation using a modification of the ‘Mitochondria : cell volume fractionator (basic)' standard pipeline. ΔψM was calculated using the Nernst equation. Because fluorescence was small in the absence of substrates, polarisation by G + M was calculated from TMRM fluorescence intensities (*F*_TMRM_) by comparison to succinate as 61 mV × log(*F*_TMRM_G + M_/*F*_TMRM_succinate_), which expresses how much less polarisation was provided by G + M than succinate, in millivolts. Polarisation by G3P was calculated by comparison to G + M as 61 mV × log(*F*_TMRM_G3P_/*F*_TMRM_G + M_), which expresses the millivolts hyperpolarisation when G3P is added in the presence of G + M. Changes in NAD(P)H autofluorescence (ΔNADH) were expressed as the observed fluorescence intensity change normalised to the population mean fluorescence before rotenone (or FCCP) addition. This mean calculation was required because some mitochondria showed very little autofluorescence. This was then converted to log_2_-fold changes to provide symmetrical distributions. Scatter plots were generated and analysed in Mathematica 13 (Wolfram Research).

### Cell culture

Fa2N-4 immortalised hepatocytes were cultured in collagen-coated dishes according to the purveyor's instructions using their proprietary media (SEKISUI XenoTech, Kansas City, KS) without cyP450 induction. Coating was performed with collagen IV (15 µg/ml in PBS) overnight. Cells were plated 2–3 days in advance of experiment into 35 mm dishes (for NAD(P)H autofluorescence) and 96-well microplates (for H_2_O_2_ production) such that on the day of the experiment monolayer density reached 80–90% confluence (e.g. 3.5 × 10^5^ and 2 × 10^4^ cells, respectively, 2 days before experiment). Before experiment, the growth medium was replaced with KRB assay buffer (135 mM NaCl, 5 mM KCl, 1 mM MgSO_4_, 0.4 mM K_2_HPO_4_, 20 mM HEPES, 1.8 mM CaCl_2_, 0.01% fatty acid-free BSA, 17 mM glucose, pH 7.4) by washing with this medium twice and then leaving 4 ml total in the 35 mm culture dish or 50 µl in 96-well plates.

### Assay of hydrogen peroxide production rates in Fa2N-4 cells

Hydrogen peroxide production rates were measured using Amplex UltraRed with a Pherastar FS (BMG) fluorescence microplate reader. Fa2N-4 cells were plated in 96-well microplates in a pattern that utilises 48 wells of the entire collagen-coated plate in a scheme that avoids cells being cultured at edge wells to prevent ‘edge effects', while leaving the other 48 wells to be used as matched background wells for each condition. Before the assay, cultures were washed with KRB assay medium twice and transferred to a 37°C air incubator for ∼10 min. Immediately before recording, KRB was completely removed from the cell assay plate, which was washed once and the medium was replaced with 50 μl per well of pre-warmed RB (reaction buffer; made of KRB containing 50 μM Amplex UltraRed, 5 U/ml HRP) pre-mixed with test compounds or vehicle for each well. Fluorescence recording was immediately started at Ex 540 nm/Em 590 nm for 25 of 80 s-cycles using 3 mm orbital well averaging in the bottom readout, kinetic mode at 37°C. Cycles 5–18 were taken for regression analysis. Background rates measured in cell-free, but otherwise identical wells were subtracted from corresponding cell–well rates. The rate of hydrogen peroxide release was expressed as % vehicle. No SOD was added to the RB as only hydrogen peroxide, but not superoxide, is freely diffusible through the cell membrane. Adding SOD impaired the signal-to-noise ratio of the recording (data not shown).

### NAD(P)H autofluorescence imaging and single-cell analysis of Fa2N-4 cells

Subcellular resolution micrographs of NAD(P)H autofluorescence were recorded using an upright Zeiss LSM 7MP multiphoton microscope, equipped with a Chameleon Vision II mode-locked laser set to 710 nm (1–1.5% of the 745 mW output power was used) and W Plan-Apochromat 20×/1.0 DIC M27 75 mm dipping water immersion objective lens and detection of emission between 420 and 480 nm. 1024 × 1024 pixels images (0.2 µm/pixel) were recorded at maximal scan speed and 8× frame averaging. Time courses of two frames, one before and one after the addition of rotenone (2 µM), FCCP (4 µM) or vehicle (DMSO 1 : 2000) were recorded using the Zeiss Multi Time Series macro. The cell monolayer was auto-focused at one *x*,*y*-position, and four other positions were imaged for analysis in each dish. Recordings were analysed in Image Analyst by rigid image registration, background subtraction and ROI-based intensity measurement. Unbiased single-cell ROIs were generated in using Cellpose 2.0 [48] with the ‘cyto2' neural network. Histograms were generated in Mathematica.

### Subcellular analysis of NAD(P)H autofluorescence in Fa2N-4 cells

The NAD(P)H autofluorescence multiphoton time courses described above were de-noised by Wiener filtering, cropped based on the Cellpose ROIs resulting in 256 × 256 pixels images of single cells, and surrounding structures were masked in Image Analyst MKII. Then, to compensate for the motility of mitochondria during the time courses, B-spline-based deformable image registration was performed in Elastix 5.0.1 [49]. Image registration was quality controlled subjectively plus by calculating Pearson’s cross-correlation coefficient between before- and after-rotenone frames, and cells with a value lower than 0.6 were discarded. The pixel-by-pixel ratio of after- over before-treatment frames was calculated, masked by mitochondrial profiles generated by Otsu’s threshold method (with a logical OR of before- and after-frames), and log_2_ of the ratios was taken for all pixels. Image histograms were saved to Excel and analysed in Mathematica using a maximum log-likelihood method for fitting the binned log_2_-fold intensity values with normal distribution(s). Mock treatments were fitted well with a single, symmetric distribution. We attribute this change of pixel intensities between the consecutive frames of the time course to motility artefacts and noise. The distribution of log_2_-fold pixel intensity changes for rotenone-treated cells was wider, and therefore we coarsely modelled it as sum of two normal distributions (rotenone-responsive and unresponsive mitochondria, with *σ* matching that of the mock-treatment modelling motility and noise effects). The fraction of mitochondria with reverse electron flow was estimated as the excess pixel count observed at significantly decreased NAD(P)H autofluorescence intensities (below 2*σ* of the mock) that could not be attributed to effects of motility and noise based on the fit above. This analysis was repeated for individual single cells (total 185 in three experiments).

### Statistics

Mitochondrial experiments were repeated using at least *n* = 3 independent mitochondrial preparations. Results are either representative traces, or means ± SEM (*n* = 3 independent experiments each the mean of at least three technical replicates), as indicated.

Experiments using cultured cells were replicated from at least three different passages (indicated by *n*) with multiple technical replicates (e.g. view fields or wells) within each experiment, which were pooled for analysis.

## Data Availability

All relevant data have been provided in the main article.

## Abbreviations

FET: forward electron transport
G + M: glutamate plus malate
G3P: glycerol 3-phosphate
I_Q_f: site I_Q_ operating during FET
I_Q_r: site I_Q_ operating during RET
mGPDH: mitochondrial isoform of G3P dehydrogenase
Pmf: protonmotive force
Q: ubiquinone
RET: reverse electron transport
S1QEL: suppressor of site I_Q_ electron leak
S3QEL: suppressor of site III_Qo_ electron leak
site G_Q_: the site of superoxide production at the Q-binding site of mGPDH
site I_F_: the site of superoxide/hydrogen peroxide production in complex I dependent in intact mitochondria only on the redox state of the flavin
site I_Q_: the site of superoxide/hydrogen peroxide production in complex I strongly dependent also on the redox state of Q and on the components of pmf
site O_F_: the site of superoxide/hydrogen peroxide production at the flavin of the 2-oxoglutarate dehydrogenase complex (OGDHC)
TMRM: tetramethylrhodamine methyl ester
